# CO_2_ Adsorption and Separation Behavior Research of Polypyrrole Under Higher Pressure

**DOI:** 10.3390/ma19214732

**Published:** 2026-11-07

**Authors:** Zhe Wang, Zhanhong Li, Ting Zhang, Zhen Wang, Jifu Huang, Hang Cao, Shupeng Li

**Affiliations:** 1 College of Ocean Engineering and Energy, Guangdong Ocean University, Zhanjiang 524088, China; wz0505@gdou.edu.cn (Z.W.);; 2 School of Mechanical Engineering, Xi’an Jiaotong University, Xi’an 710049, China; 3 State Environmental Protection Key Laboratory of Eco-Industry, Northeastern University, Shenyang 110819, China

**Keywords:** polypyrrole, high-pressure adsorption, CO_2_/N_2_ selectivity, CH_4_/N_2_ separation

## Abstract

The rise in atmospheric CO_2_ concentration makes effective carbon capture technologies increasingly important for mitigating climate change. Polypyrrole (PPy), a nitrogen-rich adsorbent material, has not been extensively studied regarding its high-pressure adsorption behavior, despite its unique advantages. In this study, we systematically investigated the adsorption and separation behaviors of PPy for CO_2_ under high-pressure conditions. High-pressure adsorption experiments demonstrated that the adsorption capacities of CO_2_, CH_4_, and N_2_ were 2.0, 0.65, and 0.41 mmol/g, respectively, at 25 °C and 15 bar. Calculations based on the IAST method indicate that PPy at high pressure exhibits adsorption selectivities of 16, 3.1, and 5.8 for CO_2_/N_2_ (15/85, *v*/*v*), CO_2_/CH_4_ (50/50, *v*/*v*), and CH_4_/N_2_ (50/50, *v*/*v*), respectively, demonstrating excellent separation performance, particularly for the CH_4_/N_2_ pair. In addition, the kinetics of adsorption were found to align with the Bangham model, suggesting that intra-particle diffusion governs the process, which is consistent with the hierarchical pore structure that facilitates rapid mass transfer. The high adsorption capacity, exceptional selectivity, and fast kinetics exhibited on the PPy adsorbent at elevated pressures underscore its potential application in industrial carbon capture fields such as flue gas treatment and natural-gas purification.

## 1. Introduction

The sustained increase in atmospheric carbon dioxide (CO_2_) concentration is widely recognized as a major contributor to global climate change and has intensified efforts to establish practical carbon capture and storage (CCS) technologies [1]. Among the available CO_2_ capture routes, adsorption-based processes have attracted considerable interest because they can potentially combine high capture efficiency, relatively low energy requirements, and convenient scale-up [2]. The effectiveness of such a process is strongly governed by the properties of the selected adsorbent. Polypyrrole (PPy) shows good environmental durability and resistance to oxidation and degradation, which favor its long-term use under demanding conditions [3]. The unique combination of electrical conductivity, flexibility, environmental stability, and biocompatibility makes PPy a versatile material with applications ranging from electronics and energy storage to biomedical and environmental technologies [4,5,6].

Ongoing research continues to expand the potential applications of PPy and its composites in diverse fields, showcasing its significance in the development of advanced materials and devices [7]. Kisiel et al. [8] synthesized PPy nanoparticle films using two straightforward template-free methods, demonstrating that the polymer films exhibit highly active and easily adaptable electrochemical properties, as determined by cyclic voltammetry, potentiometry, and the EIS method. Bansal et al. [9] conducted a study on the synthesis of self-supported PPy flexible electrodes with high resilience for the electrochemical reduction of nitrite. In the adsorption field, Wang et al. synthesized a PPy-intercalated bentonite material, denoted PPy-BT, through in situ chemical polymerization; this material efficiently removed Mo(VI) from water and retained a high regeneration capacity during consecutive adsorption–desorption cycles [10]. Comparative work on Cr(VI) uptake by PPy nanoparticles and nanotubes further showed that the nanotubular form provided stronger adsorption performance [11]. In the field of CO_2_ adsorption, existing studies on PPy and its composite adsorbents have mainly focused on ambient or low-pressure conditions [12]. Adhikari et al. [13] reported, for instance, that surfactant-free PPy achieved a CO_2_ adsorption capacity of 173.9 mL/g at 195 K. Recent studies have demonstrated that the adsorption capacity and selectivity of polypyrrole can be significantly enhanced through various modification strategies, such as doping with functional groups, composite formation with other materials, and nano-structuring [14]. These modifications can tailor the polymer’s surface chemistry and textural properties, thereby improving its interaction with CO_2_ molecules. However, the majority of these studies have been conducted at ambient or low pressures, leaving the potential for CO_2_ adsorption, especially under higher pressure conditions, relatively underexplored. Elevated-pressure operation is relevant to several industrial gas-separation scenarios, particularly natural-gas purification and other processes in which the CO_2_ partial pressure is appreciable. Understanding the CO_2_ adsorption and separation behavior of polypyrrole under these conditions is crucial for optimizing its performance in real scenarios [15].

In our previous study, the adsorption and separation of CO_2_, N_2_, and CH_4_ on pristine PPy were evaluated at atmospheric pressure, and the results identified PPy as a promising polymeric molecular sieve [16]. The linear adsorption isotherm implies a certain gas adsorption potential at elevated pressures, so the gas adsorption and separation properties of PPy at higher pressures are worth investigating. In this study, we performed the adsorption capacity tests of polypyrrole on CO_2_, N_2_, and CH_4_ gases under high pressure and explored the effect of pressure on its adsorption capacity and selectivity. In addition, we analyzed the CO_2_ adsorption kinetics of polypyrrole, which is a useful supplement to previous studies. “High pressure” here refers to pressures up to 15 bar, i.e., approximately one order of magnitude above ambient. This range is modest compared with pressures encountered in natural-gas pipelines (up to ~100 bar), but it is substantially higher than the ambient or sub-ambient pressures used in nearly all previous CO_2_ adsorption studies on PPy, and it covers the partial-pressure range relevant to flue-gas pretreatment and low-pressure natural-gas sweetening. The pseudo-first-order, pseudo-second-order, Bangham, and Elovich models are phenomenological rate expressions widely applied across pressure ranges [17]. Their transfer to high-pressure data assumes that the same elementary steps remain rate-relevant and that pressure dependence is absorbed into effective rate constants rather than altering the rate-law form [18]. This is an approximation, since elevated pressure can change both driving force and diffusivity; the models are therefore used here as comparative descriptors of uptake behavior rather than as mechanistic proof, and the fitted parameters are interpreted accordingly.

## 2. Materials and Methods

### 2.1. Adsorbent Preparation

Polypyrrole was prepared by the previously reported green oxidative polymerization route [16]. In brief, a pyrrole monomer was dispersed in aqueous medium and polymerized under controlled oxidative conditions at room temperature, without organic solvents or template agents. The resulting black precipitate was filtered, washed repeatedly with deionized water and ethanol until the filtrate was colorless, and dried under vacuum at 60 °C for 12 h. The dried powder was used directly without further purification.

### 2.2. High-Pressure Adsorption Measurements

CO_2_ (99.999%), CH_4_ (99.999%), and N_2_ (99.999%) were used as received. Prior to each measurement, the PPy sample (~0.5 g) was degassed under vacuum at 200 °C for 6 h to remove physisorbed water and air. Adsorption isotherms were measured on a gravimetric high-pressure sorption analyzer over 0–15 bar at 0, 25, and 50 °C. The temperature was controlled to ±0.1 K using a circulating bath. Each isotherm was measured in both adsorption and desorption modes to confirm reversibility. Adsorption kinetics were measured at 30, 40, and 50 °C by recording the sample mass as a function of time after stepwise introduction of CO_2_ at a fixed pressure.

### 2.3. Adsorption Isotherms and Calculation

Adsorption isotherms were simulated using the dual-site Langmuir (DSL) model [19], which is a well-established model for describing heterogeneous adsorption sites in porous adsorbents [20]:

(1) $$q=\frac{q_{1}b_{1}p}{1+b_{1}p}+\frac{q_{2}b_{2}p}{1+b_{2}p}$$

(2) $$b_{i}=b_{i0}exp(\frac{{∆H}_{i}}{RT})$$

Here, *q*_1_ and *q*_2_ correspond to the maximum capacities of site 1 and site 2 (mmol/g); *b* is the temperature-dependent adsorption equilibrium constant; ∆*H* and *T* represent adsorption heat (kJ/mol) and adsorption temperature (K), respectively; and R denotes the ideal gas constant (8.314 J·mol^−1^·K^−1^).

The ideal adsorbed solution theory (IAST) was employed to predict the selectivities of CO_2_/N_2_, CO_2_/CH_4_, and CH_4_/N_2_ [21], while the Clausius–Clapeyron equation was used to determine the isosteric heats (Q_st_) for CO_2_ and CH_4_ [22].

## 3. Results and Discussion

### 3.1. Physicochemical Properties of PPy

**Figure 1:**
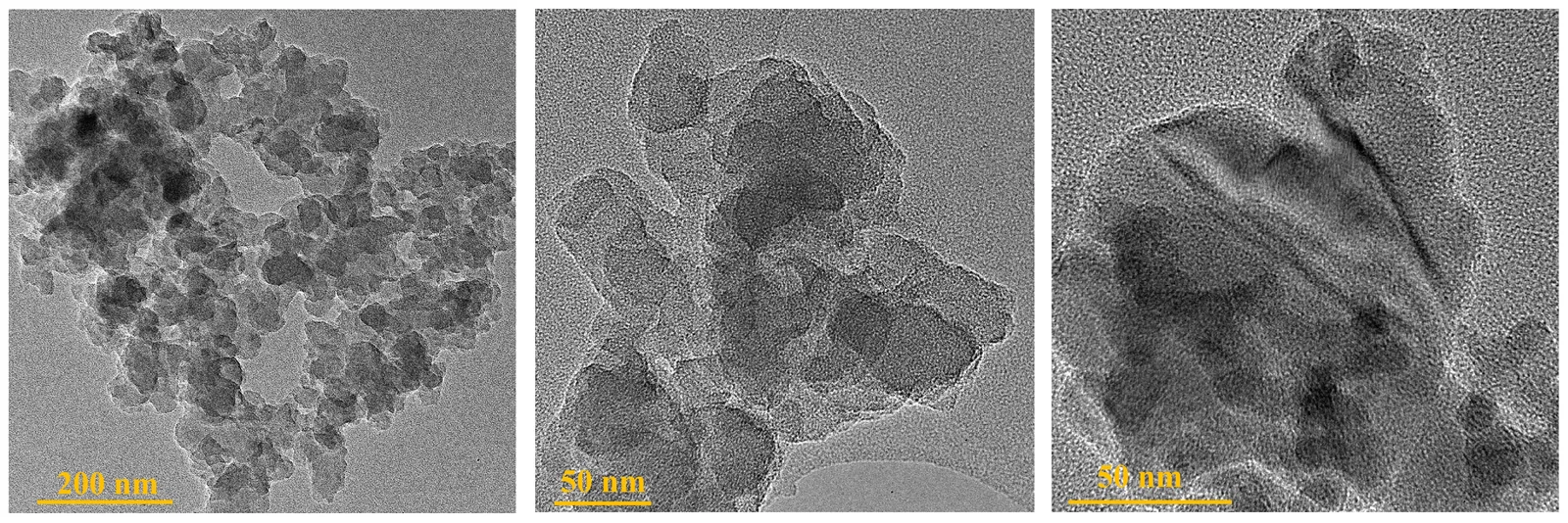
TEM images of PPy under different magnifications.

**Figure 2:**
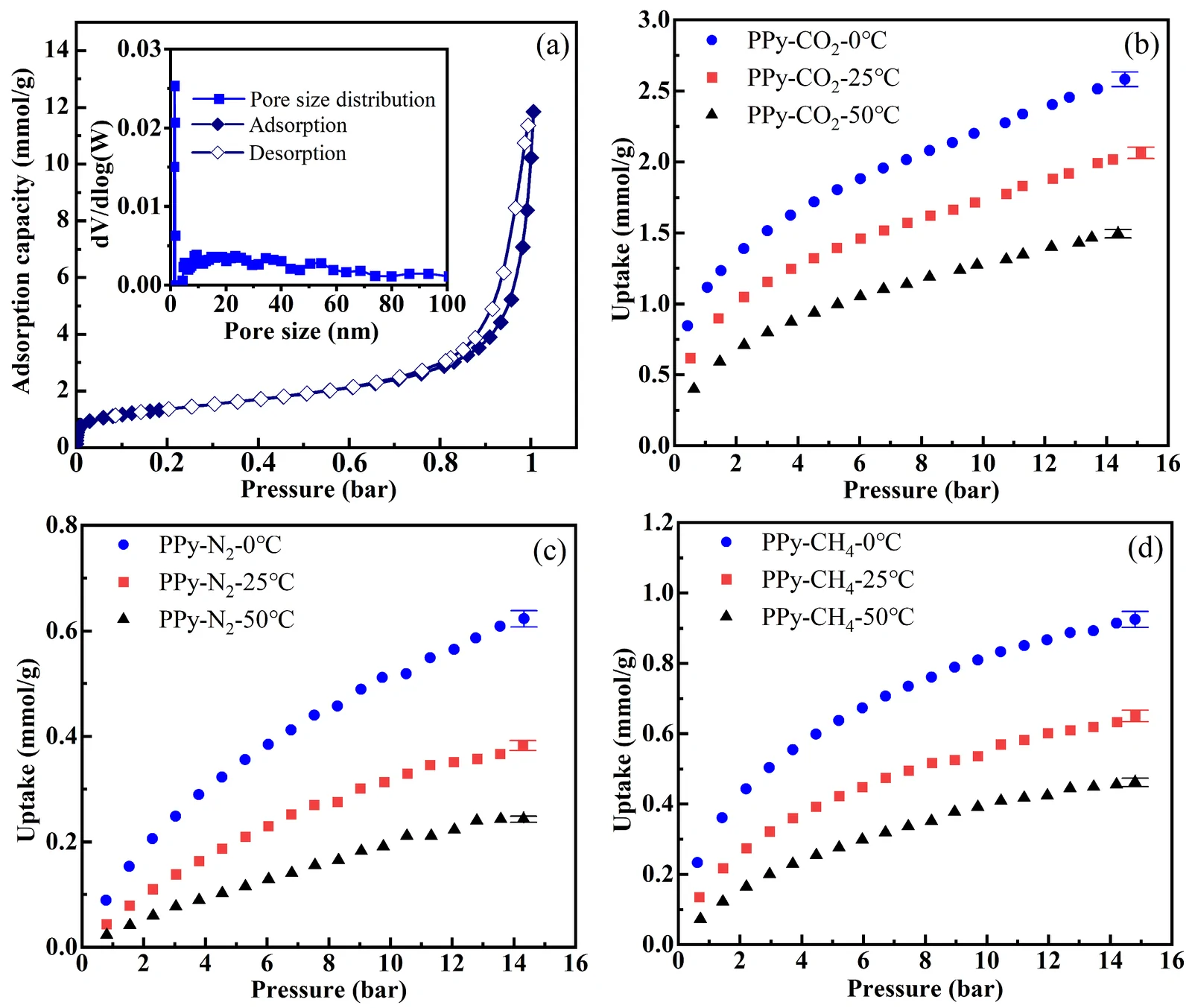
(**a**) N_2_ adsorption–desorption isotherms at 77 K (pressure axis in bar) with the pore size distribution shown in the inset; (**b**–**d**) high-pressure CO_2_, N_2_, and CH_4_ adsorption isotherms on PPy measured at 0, 25, and 50 °C; the relative standard deviation of the adsorption capacity was below 3% over the entire pressure range, and error bars in (**b**−**d**) represent one standard deviation.

Transmission electron microscopy images (Figure 1) show that the PPy synthesized in this study consists of uniformly sized spherical nanoparticles densely packed together, forming a characteristic 3D nano-aggregate structure with a primary particle diameter of approximately 50 nm. This dispersed but closely packed arrangement generates extensive external surface and numerous voids between adjacent particles [23]. The textural properties of PPy were quantified by the BET method and non-local density functional theory (NLDFT), which were used to determine the specific surface area and pore-size distribution, respectively. The measured BET surface area was 99 m^2^/g, while the total pore volume was 0.30 cm^3^/g, including 0.29 cm^3^/g assigned to mesopores. As presented in Figure 2a, the N_2_ adsorption–desorption curve exhibits a type-IV isotherm accompanied by an H3 hysteresis loop, indicating that mesopores constitute the principal pore class in PPy [24]. The pore size distribution curve further shows a concentrated pore distribution within the 2–10 nm range, along with some pore volume in a wider range (10–100 nm), suggesting that PPy is an adsorbent with a hierarchical pore structure. Additional surface-area and pore-distribution data for PPy synthesized by the same green route are provided in our published work [16]. The nitrogen-containing groups on the PPy molecular chains serve as the main active sites for CO_2_ adsorption, and the aforementioned nano-aggregate morphology and hierarchical porous structure ensure that these active sites are fully exposed on the pore surfaces [25]. The synergistic interaction between this macroscopic morphology, microscopic pore channels, and molecular sites not only provides sufficient diffusion pathways and available surfaces for gas molecules such as CO_2_, N_2_, and CH_4_ but also significantly enhances the CO_2_ adsorption capacity and capture kinetics [26], collectively constituting the key structural foundation for PPy as a high-performance adsorbent.

### 3.2. Adsorption and Separation Studies

Figure 2b–d display the high-pressure adsorption isotherms of CO_2_, N_2_, and CH_4_ on PPy at 0, 25, and 50 °C over pressures extending to 15 bar. At the same temperature and equilibrium pressure, the adsorption capacity of PPy for these gases consistently ranks as CO_2_ > CH_4_ > N_2_. The adsorption capacities obtained for CO_2_, CH_4_, and N_2_ are 2.0, 0.65, and 0.41 mmol/g, respectively, at 25 °C and 15 bar. When the pressure is varied at 15 bar, the CO_2_ uptake is 2.56 mmol/g at 0 °C, 2.0 mmol/g at 25 °C, and 1.54 mmol/g at 50 °C. The outstanding CO_2_ capture performance of PPy can be explained by two main factors. On one hand, the relatively small kinetic diameter of CO_2_ (0.33 nm) favors its diffusion within the microporous channels of PPy [27]. Based on molecular properties and thermodynamic results, we tentatively attribute preferential CO_2_ adsorption to quadrupole-N-group interactions combined with pore-diffusion effects. Further spectroscopic characterization would provide more direct molecular-scale evidence for the adsorption mechanism. In contrast, although CH_4_ molecules have a higher polarizability, their lack of a quadrupole moment limits specific interactions with the functional groups on PPy surfaces [28]. N_2_ molecules do have a quadrupole moment, but their polarizability is lower, resulting in the weakest interaction with PPy surfaces and thus the lowest adsorption capacity [29].

A nearly linear increase in the adsorption isotherms of CO_2_, CH_4_, and N_2_ on PPy is observed over the tested pressure range, and no obvious saturation plateau is evident. This indicates that PPy still has the potential for further gas adsorption capacity under higher pressure. The adsorption amount of each gas increased progressively with system pressure. At elevated pressure, the larger gas-phase chemical potential promotes pore filling and enables molecules to occupy a broader range of adsorption regions. In addition to entering the microporous domains, gas molecules may adsorb on the internal surfaces of mesopores and larger voids and may form additional adsorption layers, thereby increasing the total capacity [30]. Temperature produced the opposite effect. For all three gases, adsorption decreased substantially as the temperature rose. Higher temperature enhances molecular thermal motion and increases the probability that adsorbed molecules escape from the adsorption layer into the gas phase [31]. Consequently, heating is unfavorable for stable retention of CO_2_, CH_4_, and N_2_ on PPy, consistent with an exothermic physical adsorption process.

**Figure 3:**
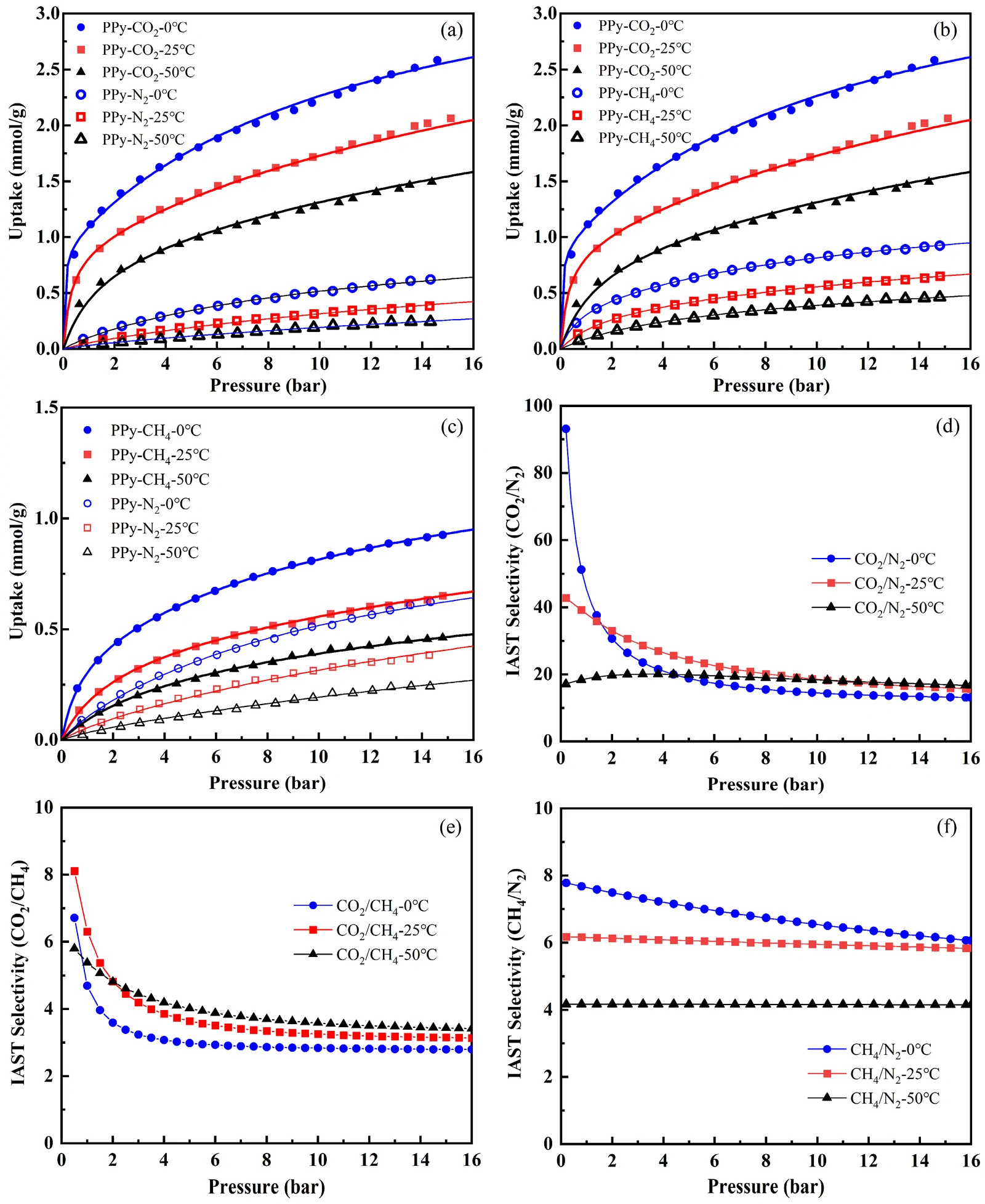
(**a**–**c**) Fitting isotherms of CO_2_, N_2_, and CH_4_ adsorption amounts; (**d**) CO_2_/N_2_ (15/85, *v*/*v*) adsorption selectivities on PPy adsorbent at high pressure; (**e**) CO_2_/CH_4_ (50/50, *v*/*v*) selectivities on PPy adsorbent at high pressure; (**f**) CH_4_/N_2_ (50/50, *v*/*v*) adsorption selectivities on PPy adsorbent at high pressure.

Using the pure-component adsorption results described above, the ideal adsorbed solution theory (IAST) was applied to estimate the mixture selectivity of PPy for CO_2_/N_2_ (15/85, *v*/*v*), CO_2_/CH_4_ (50/50, *v*/*v*), and CH_4_/N_2_ (50/50, *v*/*v*) [32]. It should be noted that IAST-predicted selectivity is calculated from pure-component isotherm data. IAST assumes ideal adsorbed-solution behavior and ignores potential adsorbate–adsorbate interactions and competitive-adsorption deviations under real mixed-gas environments. Therefore, IAST results serve as the theoretical reference. Direct binary mixed-gas breakthrough experiments are required for rigorous validation of separation performance, which will be carried out in our future research work. The gas compositions were chosen to reflect the target industrial separation scenarios. CO_2_/N_2_ (15/85, *v*/*v*) corresponds to the approximate CO_2_ concentration in coal-fired power plant flue gas, which typically contains 10–15 vol% CO_2_ with the balance being mainly N_2_. CO_2_/CH_4_ (50/50, *v*/*v*) was adopted as a benchmark composition for natural-gas sweetening, where CO_2_ must be removed from methane-rich streams; equimolar compositions are widely used in IAST studies of CO_2_/CH_4_ separation to enable direct comparison across adsorbents. CH_4_/N_2_ (50/50, *v*/*v*) represents an equimolar benchmark relevant to methane enrichment from nitrogen-rich streams such as coalbed methane and biogas upgrading, where CH_4_/N_2_ selectivity is a key performance metric. Before performing the IAST calculations, the measured isotherms were correlated with the dual-site Langmuir model. The model reproduced the experimental data closely, and the fitted parameters are summarized in Table 1. The CO_2_/N_2_ selectivity at 15 bar was calculated to be 13.2, 16.0, and 16.9 at 0, 25, and 50 °C, respectively, using the parameters obtained from fitting the adsorption isotherms. In the CO_2_/N_2_ system (Figure 3a,d), although the selectivity at different temperatures shows a downward trend with increasing pressure, it remains above 13 throughout the entire pressure range, indicating that PPy maintained substantial CO_2_/N_2_ separation ability under elevated-pressure conditions. The decline in selectivity can be related to the tendency of the CO_2_ adsorption isotherm to become saturated at high pressures, while N_2_ adsorption increases almost linearly, resulting in a shifting balance in their competitive adsorption capabilities as pressure increases [33]. Notably, the stable separation performance exhibited by PPy at high pressures allows it to demonstrate significant advantages in accommodating pressure fluctuations encountered in practical high-pressure flue gas pretreatment systems, providing important support for the application of this material in industrial carbon capture devices [34].

Based on the DSL fitting parameters, the CO_2_/CH_4_ (50/50, *v*/*v*) selectivities at 15 bar were estimated as 2.8, 3.1, and 3.4 at 0, 25, and 50 °C, respectively (Figure 3b,e). The selectivity exhibited a consistent decrease with rising pressure across all three temperatures. Additionally, an increase in selectivity was observed with increasing temperature under higher pressure conditions. This phenomenon can be attributed to the more rapid decline in the adsorption capacity of the weakly adsorbed CH_4_ with increasing temperature compared to that of the strongly adsorbed CO_2_. In the CH_4_/N_2_ (50/50, *v*/*v*) system (Figure 3c,f), the selectivity change exhibits distinct temperature dependence: at 0 °C, the CH_4_/N_2_ system exhibits relatively high selectivity that gradually decreases with increasing pressure, while at 25 °C and 50 °C, the selectivity remains relatively stable, demonstrating excellent pressure insensitivity. The stable separation behavior of PPy across a broad pressure window is evident from this characteristic, and it likely originates from the homogeneous energetic landscape of the adsorption sites and the competitive equilibrium between CH_4_ and N_2_ within the pore network [35]. It is noteworthy that at a mixture pressure of 15 bar, the CH_4_/N_2_ selectivity values of 6.1, 5.8, and 4.2 were obtained at 0, 25, and 50 °C, respectively, demonstrating excellent separation performance for these two linear molecules on the PPy adsorbent. The persistence of this selectivity during pressure variation is relevant to methane enrichment processes, including biogas upgrading, natural-gas purification, and coalbed-methane separation. The gas separation performance positions PPy as a promising adsorbent for efficient biomethane production and pipeline natural-gas treatment, underscoring its substantial potential for practical engineering applications [36].

**Figure 4:**
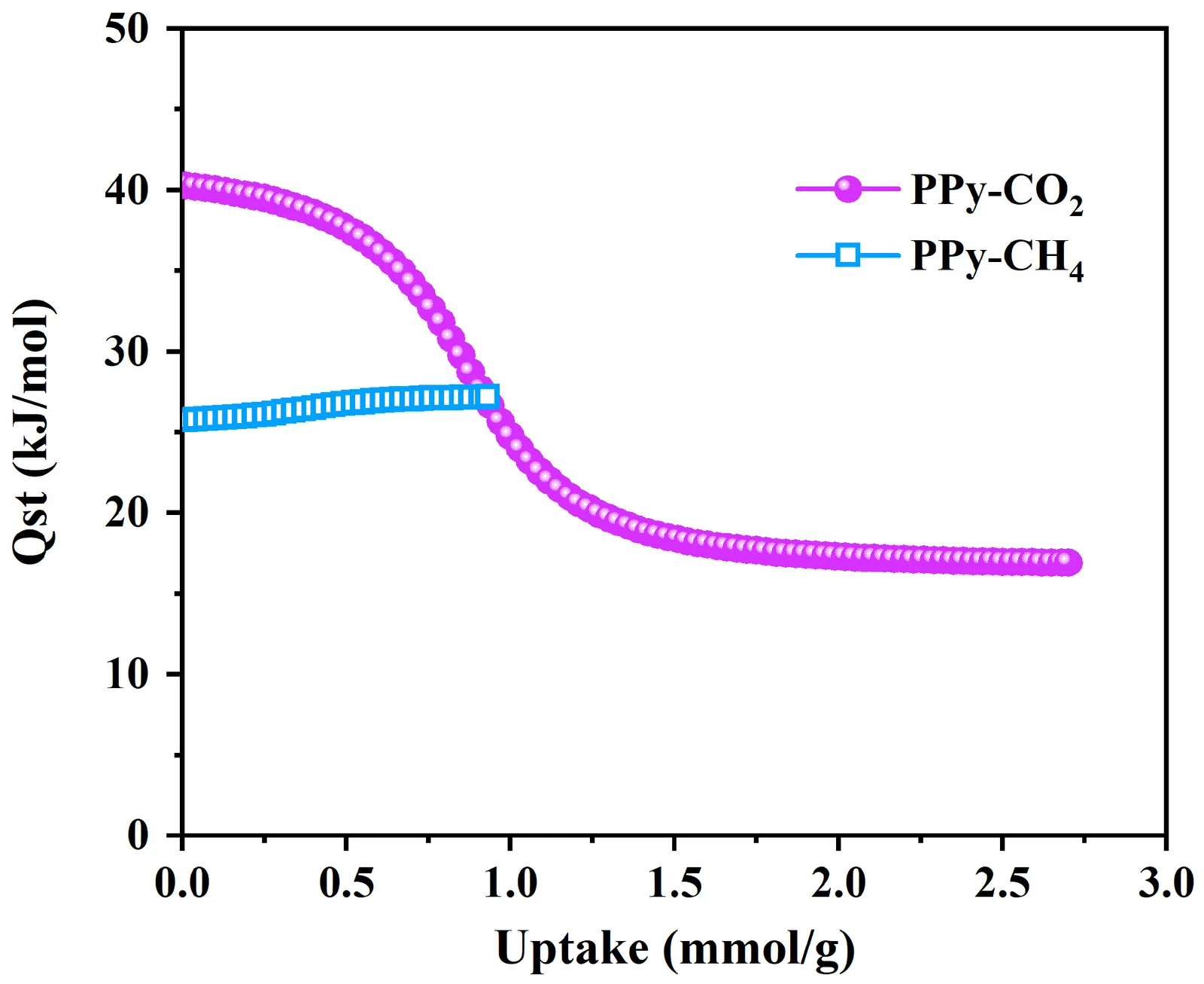
CO_2_ and CH_4_ isosteric heat of PPy adsorbent.

The isosteric heat of adsorption is an important energetic parameter for evaluating the interaction strength between an adsorbent and an adsorbate. Analysis of its loading dependence can also provide insight into the energetic origin of preferential adsorption [37]. The N_2_ isosteric heat is not shown because of the low N_2_ uptake and the near-linear isotherms, which precluded a reliable Clausius–Clapeyron analysis. As illustrated in Figure 4, the isosteric heat for CO_2_ decreased distinctly from approximately 40 to 17 kJ/mol as adsorption proceeded. This reduction indicates energetic heterogeneity on the PPy surface. At low loading, CO_2_ preferentially occupies the higher-energy regions, which may include characteristic small pores located near nitrogen-containing functional groups. With increasing loading, these favorable regions become progressively occupied and CO_2_ begins to populate more ordinary sites having lower adsorption energies [38]. In comparison, the adsorption heat for CH_4_ remains at a relatively low level and changes gently throughout, reflecting the weaker van der Waals interaction between CH_4_ and PPy. This significant difference in adsorption energy is primarily due to the diffusion advantage conferred by the smaller kinetic diameter of CO_2_ molecules as well as their strong quadrupole-specific interaction with nitrogen atoms in PPy [39]. CH_4_ interacts less strongly with the PPy surface and can therefore desorb more readily. This energetic contrast provides thermodynamic support for the preferential adsorption of CO_2_ and for the potential application of PPy in CO_2_/CH_4_ separation processes [40].

The regenerability and cyclic stability of PPy were previously evaluated in our atmospheric-pressure study on the same material, in which PPy retained its adsorption capacity and structural integrity over repeated adsorption–desorption cycles [16]. In the present study, however, high-pressure cyclic adsorption–desorption tests were not performed. The custom-built breakthrough apparatus is limited by sealing integrity, pressure tolerance, and safety considerations, which currently preclude reliable long-term cycling at 15 bar. The stability of PPy under high-pressure cyclic operation therefore remains to be established and is identified as an important objective for future work.

### 3.3. Adsorption Kinetics

The kinetic measurements were performed at 30, 40, and 50 °C, whereas the equilibrium isotherms were measured at 0, 25, and 50 °C. The difference arises from the operating limits of the two instruments: the thermogravimetric kinetic system could not be operated below 30 °C. The two data sets overlap at 50 °C, where the equilibrium capacity obtained from the Bangham fit (0.3235 mmol/g) is consistent with the value derived from the isotherm, indicating that the two measurements are mutually consistent. Kinetic model fitting helps distinguish adsorption behavior and identify mechanisms controlling the process [41]. In this study, CO_2_ uptake on PPy was evaluated using the pseudo-first-order, pseudo-second-order, Bangham, and Elovich models.

(1) The pseudo-first-order kinetics assume a linear relationship between the adsorption rate and the driving force defined by the difference between the equilibrium capacity and the amount already adsorbed [42]. The governing equation takes the following form:

$$q_{t}=q_{e}\left(1-e^{-k_{1}t}\right)$$

In this equation, q_t_ denotes the adsorbed amount at time t (mmol/g), q_e_ is the equilibrium adsorption capacity (mmol/g), and k_1_ denotes the rate constant for the pseudo-first-order kinetics [42].

(2) According to the pseudo-second-order model, the rate of adsorption is related to the squared difference between the equilibrium adsorption amount and the amount adsorbed at any time [43]. Its mathematical form is:

$$q_{t}=\frac{k_{2}q_{e}^{2}t}{1+k_{2}q_{e}t}$$

The parameter k_2_ corresponds to the rate constant of the pseudo-second-order kinetic model.

We employed the Bangham kinetic model to characterize the pore diffusion mechanism of adsorbates in the porous structure. The model equation is [44]:

$$q_{t}=q_{e}-q_{e}e^{-k_{3}t^{z}}$$

In this expression, k_3_ is the rate constant for adsorption and z is an empirical constant.

(3) The Elovich model describes adsorption kinetics where the rate declines exponentially as the adsorbed amount grows, and it is particularly suited for high-activation-energy processes [45]. The model is expressed as:

$$q_{t}=a+k_{4}\ln t$$

Here, k_4_ is defined as the rate constant for the Elovich kinetics and a is a constant.

**Figure 5:**
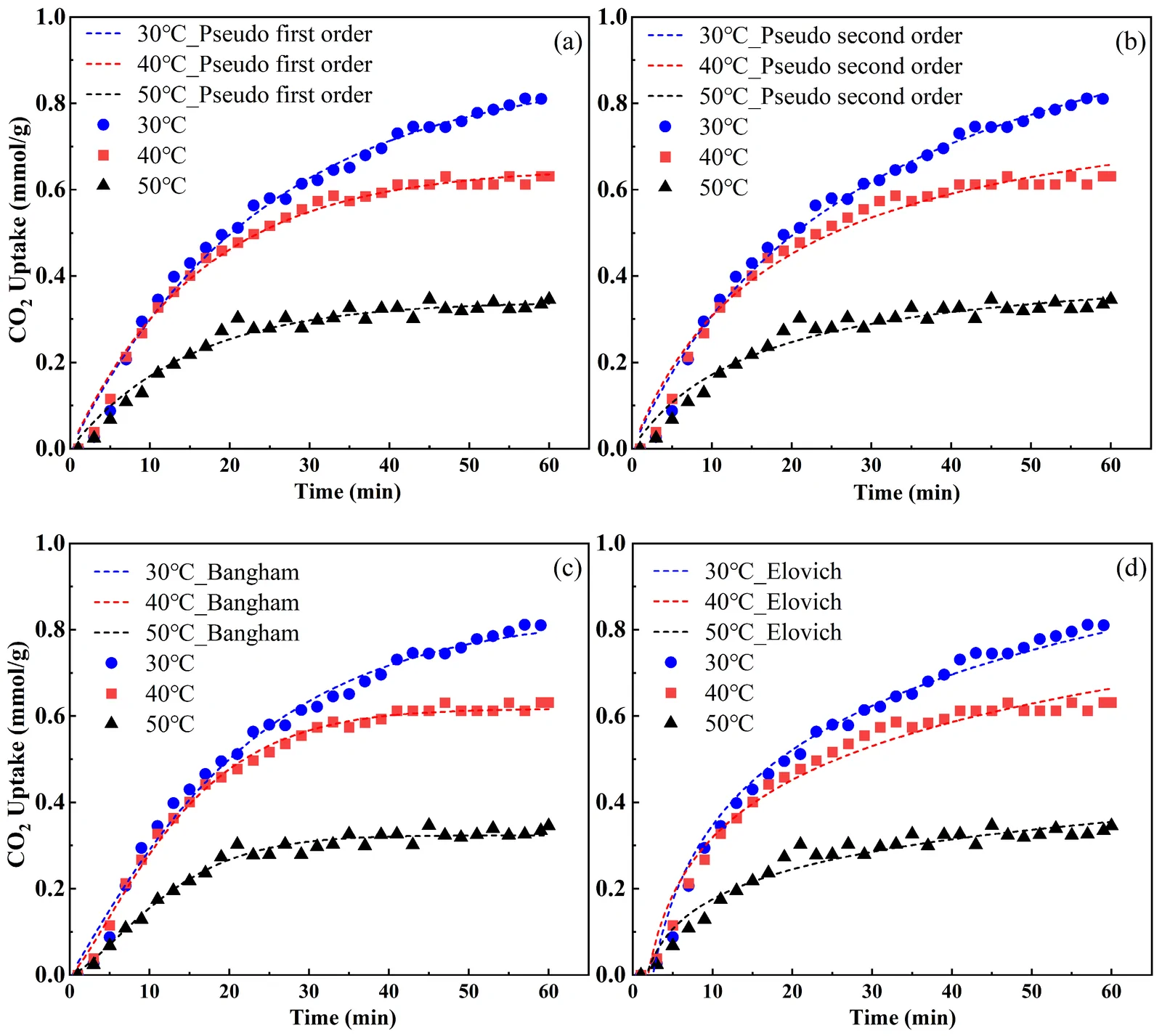
CO_2_ adsorption kinetics on PPy at 30–50 °C using (**a**) Pseudo-first-order, (**b**) Pseudo-second-order, (**c**) Bangham and (**d**) Elovich kinetic models.

Comparison of the kinetic fits for CO_2_ adsorption on PPy showed that the Bangham model produced correlation coefficients (R^2^) above 0.98 at 30, 40, and 50 °C, as presented in Figure 5 and Table 2. At all three temperatures (30, 40, and 50 °C), the R^2^ values rank in the identical order of Bangham > pseudo-first-order > pseudo-second-order > Elovich. The Bangham model yields the highest R^2^ at each temperature (0.9875, 0.9933, and 0.9827, respectively) with the highest average value of 0.988, whereas the R^2^ values of the pseudo-second-order and Elovich models decline markedly with increasing temperature (from 0.9849 to 0.9451 and from 0.9689 to 0.9350, respectively). Moreover, the fitted parameters provide consistent physical support: (i) the Elovich parameter a, which should be positive as the initial adsorption rate, was negative at all temperatures, rendering this model physically meaningless despite R^2^ values above 0.93; (ii) the pseudo-second-order model systematically overestimated the equilibrium uptake q_e_ compared with the pseudo-first-order and Bangham models; and (iii) the Bangham exponent z exceeded unity at all temperatures (1.10–1.42) and increased with temperature, which is the typical signature of intraparticle-diffusion-controlled adsorption and is consistent with the hierarchical meso-macroporous aggregate structure of PPy. The superior agreement of the Bangham equation indicates that a pore-diffusion-type description is consistent with the observed uptake behavior. However, this result should not be taken as definitive proof that intraparticle diffusion is the sole rate-controlling step [46]. The Bangham model is a phenomenological expression, and its good fit reflects the distributed nature of the adsorption sites and diffusion paths rather than a unique mechanism [47]. The open spaces between aggregated particles may serve as rapid transport channels, whereas the smaller pores provide adsorption regions with longer diffusion paths. Consequently, the measured kinetic response can reflect contributions from more than one pore scale while remaining well represented by the Bangham formulation. At 30 °C, the equilibrium capacity predicted by the Bangham model was closest to the measured value, with a difference below 0.05 mmol/g. The deviation became still smaller at the higher temperatures, indicating that the model adequately represents the measured adsorption response over the tested range. The consistent fits across the three temperatures also suggest that the principal rate behavior did not change markedly within the examined interval. These kinetic features complement previous high-pressure adsorption studies. The hierarchical pore structure of the material not only ensures adsorption capacity under high pressure, but its diffusion-controlled kinetic characteristics also guarantee separation efficiency in practical applications [48]. Especially in dynamic processes such as biogas upgrading and natural-gas purification, the rapid adsorption kinetics demonstrated by PPy provide important support for its industrial application.

Table 3 compares the high-pressure CO_2_ adsorption performance of PPy with representative adsorbents reported in the literature. Under comparable conditions (25 °C, 15 bar), PPy exhibits a CO_2_ uptake of 2.0 mmol/g, which is lower than those of high-surface-area MOFs such as MOF-808 (6.1 mmol/g at 10 bar) and its N-doped analog MOF-808@N (5.6 mmol/g) but is essentially comparable to that of sludge-activated carbon (2.1 mmol/g at 15 bar, 30 °C) [49]. Considering that PPy is a pristine polymeric material rather than a high-surface-area carbonized or framework material, this capacity is notable. More importantly, PPy combines this moderate capacity with a distinctly higher CH_4_/N_2_ selectivity (5.8) and a favorable CO_2_/N_2_ selectivity (16.0), which are rarely reported simultaneously for zeolites and activated carbons under the same conditions. MOFs such as DUT-67@N and Zr-MOF achieve higher CO_2_ uptake (5.2 and 8.1 mmol/g, respectively), but their syntheses typically involve organic ligands, metal precursors, and multi-step activation, and their high-pressure CO_2_/CH_4_ selectivity (2.2–3.6 for Zr-MOF) does not exceed that of PPy [50,51]. These comparisons indicate that, although PPy does not outperform MOFs in absolute CO_2_ capacity, it offers a balanced combination of moderate uptake, good selectivity, simple and scalable green synthesis, and intrinsic structural stability. This trade-off between capacity and selectivity, together with the low-cost and environmentally benign preparation, supports the conclusion that PPy remains a practically relevant adsorbent for high-pressure CO_2_ separation duties such as natural-gas sweetening and coalbed-methane upgrading, where selectivity and process simplicity can be as important as absolute capacity.

## 4. Conclusions

In this study, a polypyrrole adsorbent was successfully prepared through a green and feasible method, and its adsorption and separation performance for CO_2_ under high-pressure conditions was sequentially investigated. Material characterization results showed that PPy possesses a 3D agglomerated structure constructed of uniform nanoparticles, providing abundant specific surface area and effective diffusion pathways for gas adsorption. The high-pressure measurements showed that PPy preferentially adsorbed CO_2_, reaching 2.56 mmol/g at 273 K and 15 bar, while the capacities for CH_4_ and N_2_ were lower. The isotherms of all three gases continued to increase without a distinct saturation plateau in the investigated pressure region, indicating that further adsorption capacity remained available. Calculations based on the IAST for the PPy adsorbent at 15 bar and 25 °C predicted adsorption selectivities of 16, 3.1, and 5.8 for CO_2_/N_2_ (15/85, *v*/*v*), CO_2_/CH_4_ (50/50, *v*/*v*), and CH_4_/N_2_ (50/50, *v*/*v*), respectively, demonstrating excellent gas separation performance. Adsorption kinetic analysis confirmed that CO_2_ adsorption on PPy follows the Bangham model, indicating that the process is controlled by intra-particle diffusion mechanisms, which correlates well with the observed pore architecture. From a thermodynamic standpoint, the enhanced isosteric heat of CO_2_ adsorption on PPy relative to CH_4_ can be ascribed to the smaller kinetic diameter of CO_2_ and its strong quadrupole–nitrogen interactions within the PPy structure, offering a rational basis for the observed selectivity. Compared with representative MOFs, Zr-MOFs, and activated carbons under similar high-pressure conditions, PPy offers a moderate CO_2_ capacity but a superior CH_4_/N_2_ selectivity and a favorable CO_2_/N_2_ selectivity, together with a simple, green, and scalable synthesis. In conclusion, PPy exhibits a well-balanced combination of high-pressure adsorption capacity, separation selectivity, fast kinetics, and structural stability, establishing it as a promising adsorbent for CO_2_ capture and resource recovery.

## Figures and Tables

**Table 1:** Summary of DSL isotherm fitting results for CO_2_, CH_4_, and N_2_ adsorption on PPy.

Isotherms	Parameters						
	*q*_1_ (mmol/g)	*b*_10_ (1/kPa)	Δ*H*_1_ (kJ/mol)	*q*_2_ (mmol/g)	*b*_20_ (1/kPa)	Δ*H*_2_ (kJ/mol)	R^2^
CO_2_	2.96	1.12 × 10^−5^	−20.3	0.89	4.66 × 10^−9^	−50.6	0.9967
CH_4_	0.40	1.79 × 10^−5^	−25.6	1.08	4.15 × 10^−7^	−27.3	0.9995
N_2_	1.07	3.99 × 10^−6^	−22.6	0.04	5.25 × 10^−3^	−12.8	0.9983

**Table 2:** Comparison of kinetic model fitting results and parameters for CO_2_ adsorption on PPy.

Kinetic Model	Parameters	30 °C	40 °C	50 °C
Pseudo-first-order	qe (mmol/g)	0.8799	0.6520	0.3419
	k_1_	0.0416	0.0616	0.0678
	R^2^	0.9866	0.9828	0.9651
Pseudo-second-order	qe (mmol/g)	1.2443	0.8535	0.4401
	k_2_	0.0264	0.0658	0.1452
	R^2^	0.9849	0.9684	0.9451
Bangham	qe (mmol/g)	0.8355	0.6173	0.3235
	k_3_	0.0340	0.0309	0.0249
	z	1.0979	1.2919	1.4196
	R^2^	0.9875	0.9933	0.9827
Elovich	a	−0.2329	−0.1252	−0.0543
	k_4_	0.2519	0.1928	0.0999
	R^2^	0.9689	0.9633	0.9350

**Table 3:** High-pressure CO_2_ adsorption performance of PPy and representative adsorbents.

Adsorbent	T (°C)	P (bar)	CO_2_ Uptake (mmol/g)	Selectivity	Ref.
PPy (this study)	25	15	2.0	CO_2_/N_2_ = 16.0; CO_2_/CH_4_ = 3.1; CH_4_/N_2_ = 5.8	This study
MOF-808	25	10	6.1		[50]
MOF-808@N	25	10	5.6		[50]
DUT-67@N	25	10	5.2		[50]
Zr-MOF	0	9.88	8.1	CO_2_/CH_4_ = 2.2–3.6	[51]
Sludge-activated carbon	30	15	2.1		[49]

## Data Availability

The original contributions presented in this study are included in the article. Further inquiries can be directed to the corresponding author.

## Abbreviations

The following abbreviations are used in this manuscript:

CO_2_	carbon dioxide
CCS	carbon capture and storage
PPy	polypyrrole
DSL	dual-site Langmuir
IAST	ideal adsorbed solution theory
NLDFT	non-local density functional theory
